# Molecular Docking and Dynamics Simulation Revealed the Potential Inhibitory Activity of New Drugs against Human Topoisomerase I Receptor

**DOI:** 10.3390/ijms232314652

**Published:** 2022-11-24

**Authors:** Francesco Madeddu, Jessica Di Martino, Michele Pieroni, Davide Del Buono, Paolo Bottoni, Lorenzo Botta, Tiziana Castrignanò, Raffaele Saladino

**Affiliations:** 1 Department of Computer Science, “Sapienza” University of Rome, Piazzale Aldo Moro, 5, 00185 Rome, Italy; 2 Department of Ecological and Biological Sciences, Tuscia University, Largo dell’Università snc, 01100 Viterbo, Italy

**Keywords:** camptothecin, human topoisomerase I, molecular docking, molecular dynamics, ligand-receptor interaction

## Abstract

Human Topoisomerase I (hTop1p) is a ubiquitous enzyme that relaxes supercoiled DNA through a conserved mechanism involving transient breakage, rotation, and binding. Htop1p is the molecular target of the chemotherapeutic drug camptothecin (CPT). It causes the hTop1p-DNA complex to slow down the binding process and clash with the replicative machinery during the S phase of the cell cycle, forcing cells to activate the apoptotic response. This gives hTop1p a central role in cancer therapy. Recently, two artesunic acid derivatives (compounds **c6** and **c7**) have been proposed as promising inhibitors of hTop1p with possible antitumor activity. We used several computational approaches to obtain in silico confirmations of the experimental data and to form a comprehensive dynamic description of the ligand-receptor system. We performed molecular docking analyses to verify the ability of the two new derivatives to access the enzyme-DNA interface, and a classical molecular dynamics simulation was performed to assess the capacity of the two compounds to maintain a stable binding pose over time. Finally, we calculated the noncovalent interactions between the two new derivatives and the hTop1p receptor in order to propose a possible inhibitory mechanism like that adopted by CPT.

## 1. Introduction

Human DNA Topoisomerase I is a ubiquitous enzyme that relaxes DNA supercoiling through two transesterification reactions [1]. The biological activity of hTop1p is essential to ensure the normal course of several cellular processes such as DNA replication, transcription and recombination [2]. In the first transesterification reactions, hTop1p catalyzes a transient single-strand break at the target DNA sequence forming a 3′-phosphotyrosine linkage between the catalytic tyrosine (Tyr723) and the nicked DNA [3]. This covalent intermediate is generally known as the “cleavage complex” in which a random number of supercoils are removed [4]. The second transesterification reaction is used by the enzyme to complete the relegation process by inverting the tyrosine-DNA bound [5]. Structural data report that hTop1p consists of four domains: the N-terminal domain, the core domain containing all the active site residues apart from the catalytic tyrosine, the linker domain, and the C-terminal domain containing the catalytic tyrosine [6]. Human DNA Topoisomerase I is the molecular target of the chemotherapeutic drug camptothecin (CPT) [7]. CPT is a reversible hTop1p inhibitor that increases the “cleavage complex” lifetime by slowing the relegation process. This makes the impact, during the S-phase, between the blocked hTop1p-DNA complexes and the replication machinery the cause of DNA damage and apoptosis response [8]. The ability of the trapped “cleavage complexes” to lead to cell death gives hTop1p a central role in cancer therapy [9]. Artesunic acid is the water-soluble hemisuccinate derivative of artemisinin, a natural sesquiterpene lactone extracted from the Chinese herb *Artemisia annua* L., with high antimalarial activity [10]. Several studies have also reported the promising activity of these compounds toward different types of cancer cell lines, in vitro and in vivo [11]. In particular Botta et al. [12] showed that novel hybrid and dimeric derivatives of artesunic acid and artemisinin can inhibit the growth of yeast cells overexpressing human topoisomerase 1 and its enzyme activity in vitro. In that paper, the authors experimentally demonstrated that these derivatives also exhibited antitumor activity in melanoma cell lines superior to camptothecin. In a further work [13], a library of hybrid compounds and dimers based on the natural artemisinin scaffold was synthetized and the role of each new compound was studied for its antitumor activity on a cervical cancer cell line (HeLa) and three complementary metastatic melanoma cancer cell lines (SK-MEL3, SK-MEL24, and RPMI-7951). These last two experimental papers demonstrated the potential efficacy of the new hybrid and dimeric derivatives of artesunic acid and artemisinin by showing the inhibition of hTop1p 1 activity in their presence. In particular, two (compounds **6** and **7**, hereinafter referred to as **c6** and **c7**) among ten ligands showed a very high inhibition activity versus htop1p. Thus, through enzymatic assays, the effect of all the studied ligands on h1top1p was known but not the underlying interaction mechanisms explaining their inhibitory capacities.

Therefore, we considered the latter two works [12,13] as an experimental starting point for further computational investigations and chose compounds **6** and **7** as ligands of interest. Here, the questions we try to answer through simulation and computational analysis are: why do two of the new artemisinin-based hybrids and dimers have a particularly high inhibitory activity against human topoisomerase 1? What types of interactions do these two compounds form with the receptor? How long-lasting and how stable are the interactions with the receptor? We chose, as the object of study, the ligand-receptor complexes formed by human topoisomerase 1 complexed with DNA and each of the two compounds (**c6** and **c7**) with greater inhibitory activity from [12,13]. Our aim was therefore to understand the ligand-receptor interaction mechanisms of the two chosen compounds to better explain their great inhibitory activity towards htop1p. Through docking and molecular dynamics analysis, we were able to obtain an “all atoms” picture of the interactions involved in our two solvated complexes. Hence, we investigated, in silico, the inhibitory activity against hTop1p of the two new artemisinin-based hybrid derivatives (**c6** and **c7**) obtained by chemical coupling of artesunic acid and a phytochemical product known for its anticancer activity, the tyrosol [12].

**Figure 1:**
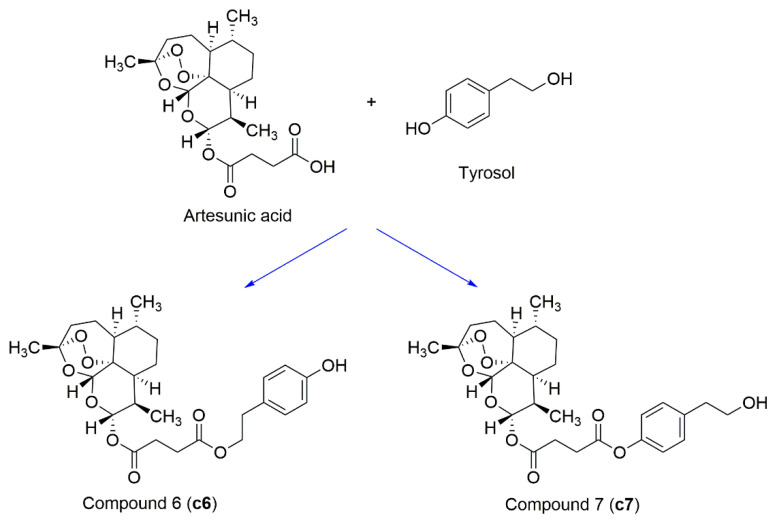
Chemical structure of **c6** and **c7** compounds.

Complex solvated macromolecular systems have been extensively investigated in recent decades through in silico techniques, such as molecular dynamics simulation [14,15,16,17,18]. In particular, the study of ligand-receptor binding interaction was addressed through virtual screening, docking, and molecular dynamics techniques [19,20]. Here, in this study, we investigated in silico the inhibitory activity against hTop1p of two new artemisinin-based hybrid derivatives (compounds **6** and **7**) obtained by chemical coupling of artesunic acid and a phytochemical product known for its anticancer activity, tyrosol (Figure 1).

**Figure 2:**
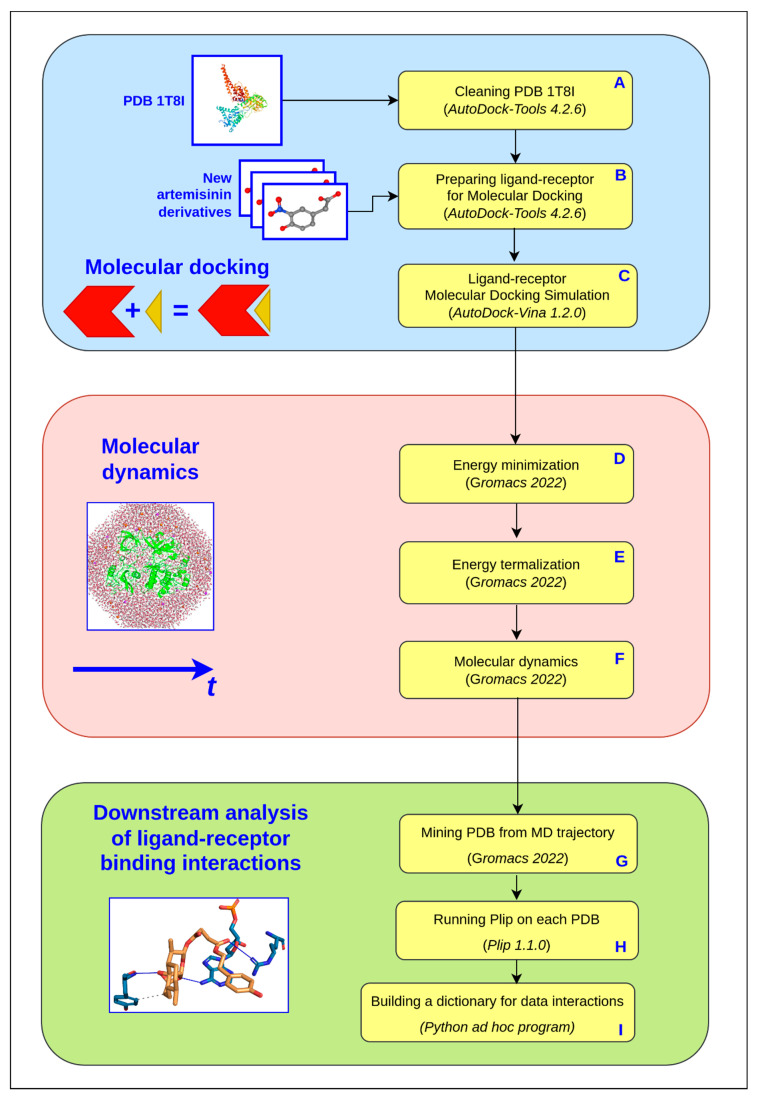
The protocol workflow implemented for the molecular docking simulation, the molecular dynamics simulation and the downstream analysis. In detail: the first phase involves molecular docking simulations (steps A–C) using Autodock Tools and Autodock Vina. This phase involves: (A) cleaning the receptor (1T8I), (B) preparing the receptor and the ligand structure (in pdbqt format), and setting the configuration file for docking, (C) running the molecular docking simulations, one for each ligand-receptor complex. The molecular dynamics steps (D–F) involve the use of Gromacs to (D) estimate the energy minimization (executed by the steepest descent and the conjugated gradient methods), (E) run MD simulations in the Isothermal–Isobaric (NPT) ensemble to equilibrate pressure and temperature, (F) run MD simulations (compute the potential interactions and solve Newton’s equations of motion). Finally, in the last phase of the protocol, downstream analysis of the ligand-receptor interactions was performed by (G) extracting each PDB from the MD trajectory, (H) running PLIP on each PDB and (I) finally building a dictionary containg all the ligand-receptor binding interactions over time.

To understand in detail the computational study presented in this article, Figure 2 shows the workflow of the implemented simulation and analysis pipeline.

First of all, we downloaded the crystallographic structure from the Protein Data Bank, 1T8I, containing the human DNA enzyme topoisomerase I (70 Kda) in the complex with the poisonous camptothecin and covalent complex with a DNA duplex of 22 base pairs. This structure was cleaned (step A in the molecular docking phase) with the AutoDockTools eliminating the camptothecin to create space in the receptor pocket for the subsequent docking with the ligands in the study. The Smile format of each ligand was converted in 3D and prepared (step B in the molecular docking phase) with the AutoDockTools for molecular docking simulation. The protocol used for preparing both ligand and receptor for molecular docking simulation in steps A and B has been extensively described elsewhere [21]. The last, C step of molecular docking simulation, was carried out with AutoDock Vina, one of the fastest and most widely used open-source programs for molecular docking. Both the receptor and each ligand were passed to the software, as inputs in pdbqt format to get the best ligand poses. In Table 1 we listed the 2D ligand structure, the Smile string, the binding affinity of the best pose, and the reference of the article where the ligands were syntetized and tested. As it can be noticed from molecular docking results (Table 1), two ligands, **c6** and **c7**, have the greatest binding affinity, confirming their putative role as alternative inhibitors to camptothecin. These results were also confirmed by previous experimental works [12,13]. Therefore, the molecular docking analysis was performed to verify the compounds’ access to the enzyme-DNA interface and further confirm the experimental data related to the enzymatic assays. In the second phase, we performed a molecular dynamics simulation of both ternary complexes solvated in saline solution, htop1p-c6 and htop1p-c7. MD is a computational technique that simulates the dynamic behavior of molecular systems as a function of time, dealing with all the entities involved (ligand, protein, provided water if explicit) as flexible. In our simulations, the complexes with the ligand best poses obtained in the previous molecular docking phase were used as the starting MD conformations. To propose an inhibitory mechanism of these two novel hybrid derivatives of the artesunic acid, we performed all-atoms MD simulation of 1 μs duration for both ternary complexes and then evaluated the stability of the two new artemisinin derivatives. Details of the MD used protocol for energy minimization (step D), energy thermalization (step E) and molecular dynamics (step F) were provided in the method paragraph. As a result of the MD phase we got the trajectories of both the ternary complexes solvated in saline solution, htop1p-c6 and htop1p-c7. These trajectories were the input data for the analysis described in the third phase. This phase of the protocol was designed to analyze ligand-receptor interactions over time. The first step, step G, regarded the extraction of conformations of ternary complexes solvated in saline solution from MD trajectory every 10 ps. In the second step, step H, of the downstream analysis (third protocol phase), the conformations output of the previous step was passed as input to the software PLIP, a protein-ligand interaction profiler, to detect all the relevant ligand-receptor binding interactions. To manage and classify all the ligand-receptor binding interactions detected in the previous step, we developed and used an ad hoc python program (step I) capable of building a python dictionary of the detected data, associating a time frame with each of them. This way we were able to count the duration in time of each bond, allowing the classification by type (hbonds, pi-stacks, etc.) and the identification by tuples of atoms involved.

## 2. Results

### 2.1. In Silico Molecular Docking

New artemisinin-based hybrid and dimer derivatives were previously tested in vitro and in vivo to evaluate their inhibitory activity against hTop1p [12,24]. In light of the experimental data, Botta et al. proposed a similar inhibitory mechanism between these artemisinin derivatives and CPT, in particular, inhibitory activity was experimentally attributed to compounds **c6** and **c7**. To perform molecular docking analyses of all the compounds investigated in [12,13], we started by studying the structure of human DNA topoisomerase I in complex with camptothecin venom (CPT) and in covalent complex with a 22-base-pair DNA duplex (PDB ID: 1T8I) [25]. In the X-ray crystal structure of 1T8I, CPT is intercalated at the DNA cleavage site between the −1/+1 base pairs and carries out its inhibitory activity by making H-bonding interactions with Arg364 and Asp533 at the distance of 2.9 Å and 3.4 Å, respectively [26]. We validated the docking protocol: the reversible CPT inhibitor was extracted from the crystal structure and then reinserted into the receptor. We found that the docked conformation of CPT overlapped with the X-ray crystal pose with a root mean square deviation (RMSD) of 2.0 Å. Once the system was validated, we performed molecular docking analyses using the CPT-deprived 1T8I complex as receptor and, as ligands, (i) the new synthetized artemisinin derivatives described in [13], (compounds **c0**–**c7**), (ii) the camptothecin (CPT), (iii) other three compounds (CPT-derived) [22,23]. AutoDockTools [27,28] and Autodock Vina [28,29] software were used for all the docking in silico experiments. The scoring functions of the Autodock Vina software include repulsions, hydrophobes, hydrogen bonds and the number of rotatable active bonds between heavy atoms as energy terms. All the molecular docking results in terms of binding affinity with the receptor were listed in Table 1. As it can be noticed, between the artemisinin derivatives, the two compounds **c6** and **c7** showed the highest binding affinity (respectively 9.1 and 8.7 kcal/mol), thus explaining and confirming their inhibitory capacity already tested in [13]. The compound Topotecan, CPT-derived, also showed a high binding affinity (8.7 kcal/mol), but, as described in the article [23], it resulted highly toxic. For both compound **6** and compound **7**, the sesquiterpene lactone portion, also called the artemisinin portion, is located in the accessory pocket of human DNA topoisomerase I, characterized by the presence of the amino acid Met428, as previously suggested by Botta et al. [13]. Instead, both active portions of the phytopharmaceutical are located in the CPT identification site. To ensure the reproducibility of the study we created a public project on figshare containing all the input and output data of molecular docking simulations (see the link of the project at “Data Availability Statement”).

Unfortunately, the molecular docking simulation technique is subject to some limitations. As described in [21], the main limitations concern the lack of explicit waters in the simulation and a static conformation result of the complex. For this reason, we studied the two complexes formed by the most interesting compounds (higher binding affinity and inhibitory activity with the receptor) with the molecular dynamics technique that allowed us to study the evolution over time of the solvated system.

### 2.2. Classical Molecular Dynamics Simulation

**Figure 3:**
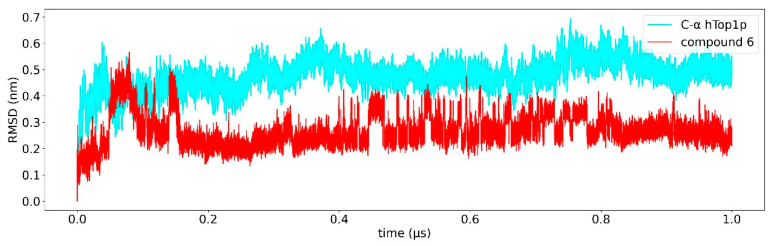
Root mean square deviation (RMSD) relative to 1 us of simulation. The RMSD of Cα of the protein is shown in cyan, the RMSD of compound **c6** is shown red.

**Figure 4:**
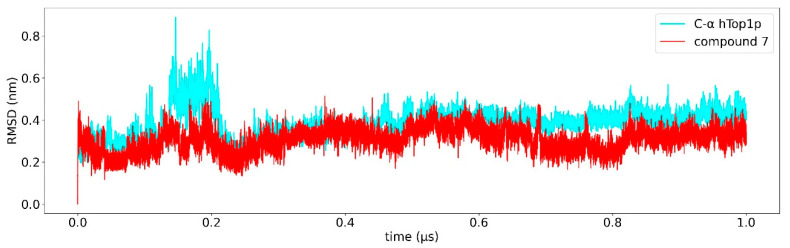
Root mean square deviation (RMSD) relative to 1 us of simulation. The RMSD of Cα of the protein is shown in cyan, the RMSD of compound **c7** is shown red.

**Figure 5:**
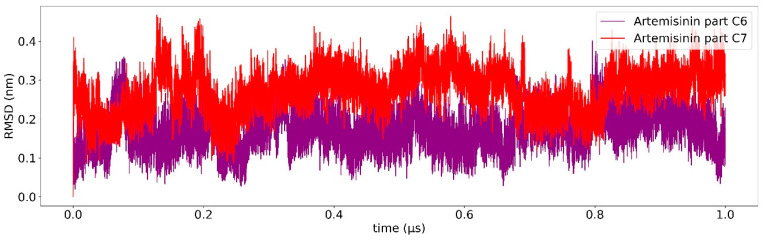
Root mean square deviation (RMSD) relative to 1 us of simulation. The RMSD of the artemisinin part of compound **c6** is shown in purple, the artemisinin part of compound **c7** is shown in red.

**Figure 6:**
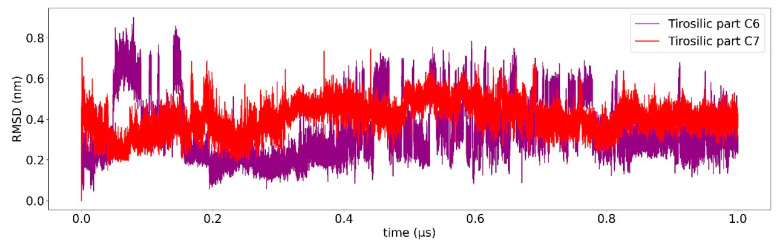
Root mean square deviation (RMSD) relative to 1 us of simulation. The RMSD of the tyrosolic part of compound **c6** is shown in purple, the tyrosolic part of compound **c7** is shown in red.

Classical MD simulation of 1T8I with the best docked conformations of compounds **6** and **7** were carried out for 1 us to study the overall stability of these compounds. The average RMSD of compounds **6** and **7** as a function of time was found to be 0.3 nm (Figure 3 and Figure 4), suggesting high binding stability, in agreement with what was previously observed in the docking study. Greater stability of compound **7** is observed compared to compound **6**. In addition, the RMSD of both ligands was studied in detail. Figure 5 and Figure 6 show the RMSD as a function of time for the atoms of the:

1. artemisinin part of both ligands (Figure 5), and
2. tirosolic part of both ligands (Figure 6).

As shown in both images, the artemisinin part of the ligand, linked to the accessory pocket of hTop1p, contributes more to the stability of the system, to the detriment of the tyrosol part which assumes different binding conformations.

We also focused our attention on evaluating the flexibility of amino acid residues (RMSF) present in the core domain of hTop1p (amino acids 215 to 635), which are the main constituents of the binding site for the two compounds.

High RMSF values indicate high flexibility of residuals during simulation.

**Figure 7:**
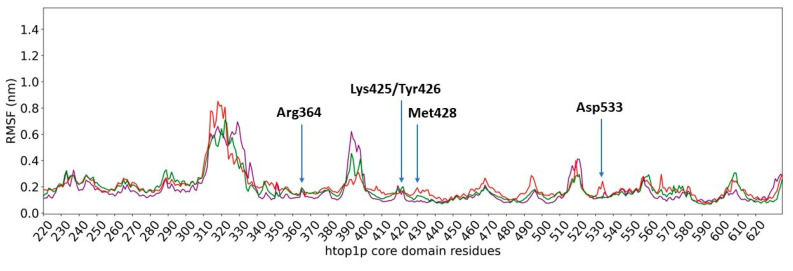
RMSF (root mean square fluctuation) of the core domain of hTop1p. In purple: RMSF Htop1p core domain in complex with compound **6**. In red: RMSF Htop1p core domain in complex with compound **7**. In green: RMSF Htop1p core domain. In evidence the root mean square fluctuation of the Cα of the amino acids Arg364, Lys425, Tyr426, Met428 and Asp533, important in constituting the binding site of compounds **c6** and **c7**.

As shown in Figure 7, no significant difference is observed when comparing the RMSFs of the core domain of hTop1p as a whole with **c6** (in purple) and with **c7** (in red). In both cases, a high flexibility is found in the core domain region that extends from residue 310 to residue 340, while the remaining regions show controlled fluctuations during simulation. The RMSF of the core domain of hTop1p in the absence of ligands (in green) confirms the fluctuations observed for this domain in the presence of **c6** and **c7**, demonstrating that neither of the two derivatives of artesunate causes structural alterations.

### 2.3. Ligand Receptor Binding Interactions as a Function of the Time

By running the MD-ligand-receptor pipeline, we identified the ligand-receptor interactions present for most of the simulation time. These were classified by bond type for both compounds. As shown in Table 2, hydrogen bonds and hydrophobic interactions are present for most of the simulation and contribute significantly to the stabilization of the system. Albeit for less time, water bridges and pi-stacking interactions between atoms also occur.

**Figure 8:**
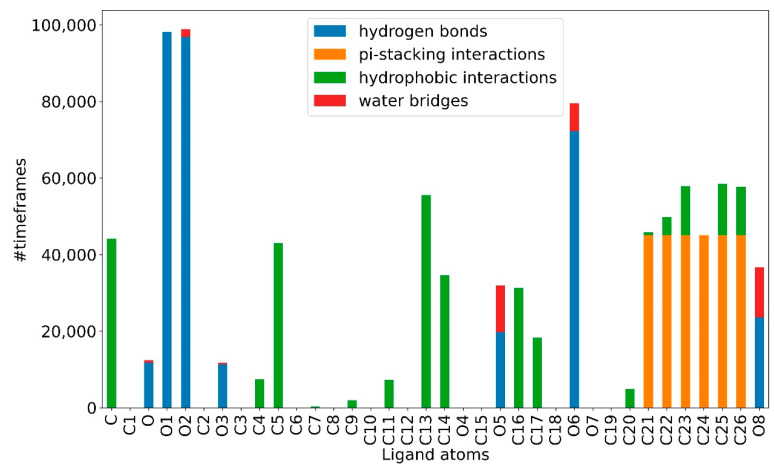
Type of bond established by a single atom of the **c6** ligand.

**Figure 9:**
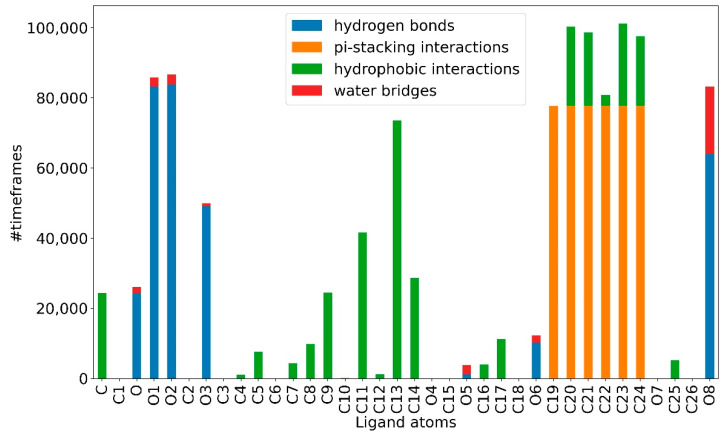
Type of bond established by a single atom of the **c7** ligand.

At the atomic level, for the atoms of the artemisinin part of both compounds, it is possible to observe, for most of the simulation, a greater propensity to form hydrogen bonds and stable hydrophobic interactions. While the atoms of the tyrosolic portion are more engaged in water bridges and pi-stacking interactions for fewer time frames, making this portion less stable (Figure 8 and Figure 9). This appears to agree with the previous RMSD results. It can be seen from Figure 8 and Figure 9 how, again, compound **c7** appears to be more stable than compound **c6** as it can establish strong bonds in greater number and that last longer.

During the analysis of the interactions, the focus was on the amino acids and nucleotide bases most involved in noncovalent interactions with 1T8I, particularly the amino acid residues Arg364 and Asp533. The residues most involved in noncovalent interactions with **c6** include the amino acids Arg364 and Tyr426 and the nucleotide bases DT9, DA113 and DA114. In particular, the amino acid Tyr426 and the nucleotide bases DT9, DA113 and DA114 play key roles in stabilizing the artemisinin portion of the compound (Table 3). Of particular note are the O1-Tyr426 and O2-DA113 interactions, which remain constant in 98% and 93% of the time simulations, respectively. Oxygen O6, on the other hand, is mainly responsible for the key interactions observed with the amino acid Arg364. Such O6-Arg364 interactions are kept constant for 73% of the trajectory snapshots. As for **c7**, it shows a common behavior with **c6** in the ability of the artemisinin portion to form weak interactions with the amino acid Tyr426 and the nucleotide bases DT9, DA113 and DA114. Again, a strong propensity of the O1 atom of **c7** to form interactions with Tyr426 is observed, while O2 and O3 participate in the stabilization of **c7** (Table 4).

**Figure 10:**
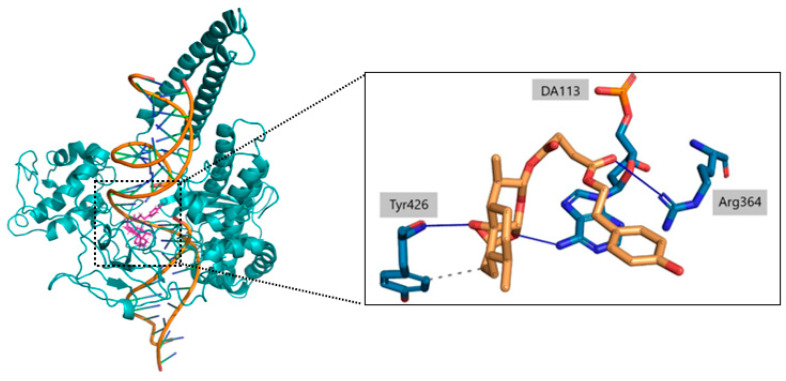
Right: 3D structures of compound **c6** housed within the receptor pocket. Left: Highlighted are the atoms most involved in the formation of noncovalent interactions (hbonds in blue, hydrophobic bonds with dashed line) with the amino acids and nucleotides of the 1T8I receptor (belonging to the core-subdomain I portion of the protein and with the DNA double helix).

**Figure 11:**
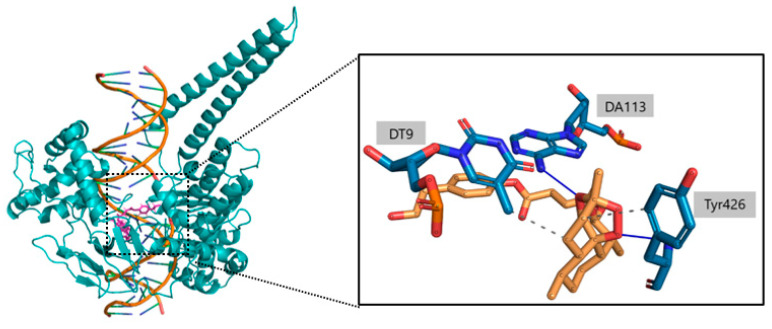
Right: 3D structures of compound **c7** housed within the receptor pocket. Left: Highlighted are the atoms most involved in the formation of noncovalent interactions (hbonds in blue, hydrophobic bonds with dashed line) with the amino acids and nucleotides of the 1T8I receptor (belonging to the core-subdomain I portion of the protein and with the DNA double helix).

In Figure 10 and Figure 11 we reported the chemical structure of the compounds and highlighted the main residues responsible for direct bonds with the receptor.

## 3. Discussion

A computational biology workflow was applied to the study of novel artesunate derivatives [12] to propose a possible inhibitory mechanism against the hTop1p alternative respect to camptothecin [13]. From experimental evidence [13], an in silico molecular docking analysis was conducted in order to evaluate the binding conformation between the novel artesunate compounds and hTop1p CPT-deprived. This analysis highlighted that two ligands, compounds **c6** and **c7**, showed the highest binding affinity to hTop1p with respect to the other evaluated ligands, including the camptothecin. Both these compounds showed also in their molecular docking best pose, the ability of the artemisinin portion to dock in the accessory pocket of hTop1p while the bioactive counterpart is laid down in the CPT recognition site. A molecular dynamics simulation, of the duration of 1 μs, was conducted on the solvated complexes hTop1p-c6 and hTop1p-c7 to assess the stability of the binding conformations over time. The two RMSDs of Cα, for the solvated hTop1p (CPT-deprived) receptor in complex with **c6** and **c7**, were stable for all the simulation time (Figure 3 and Figure 4). Also each compound in the complex was stable (only 2–3 Ǻ on average from the starting configuration) for all the simulation time, showing no major alterations of the starting bond conformation. The movements observed during the molecular dynamics simulation occur mainly at the expense of the active phytochemical portion of the two compounds (tyrosolic portion of both compounds), which assumes different binding conformations, while the artemisinin counterpart shows excellent binding stability in the accessory pocket of hTop1p (Figure 5 and Figure 6). The RMSF analysis of the core domain of hTop1p shows that some of the key amino acid residues, exhibit low and controlled fluctuations in the formation of the binding pocket for the two compounds. This finding suggests the possible involvement of these residues in stabilizing the binding conformation of the two compounds through the formation of noncovalent interactions. Finally, detection of the noncovalent interactions of the **c6**-1T8I and **c7**-1T8I complexes was performed to evaluate the receptor residues most involved in the interactions and suggest a possible CPT-like mechanism of inhibition. For both compounds, the major binding contribution observed in the stabilization of the artemisinin portion is at amino acid Tyr426 and nucleotide bases DA113 and DA114. Table 3 and Table 4 show a greater propensity of the Artemisinin part to establish a greater number of hydrophobic interactions. This confirms the excellent stability of this portion observed by the analysis of RMSD.

As for the tyrosol portion, a lower propensity to form non-covalent interactions is observed for c6 than for **c7**, for which an ability to form multiple hydrogen bonds through the terminal OH group was observed. This difference underlies the greater movements observed in the RMSD of the tyrosol portion of c6 compared to **c7**. Furthermore the ability of **c6** and **c7** to form long-lasting interactions over time with the amino acids Arg364 and Asp533, which represent the key residues for carrying out the inhibitory action of CPT (chromophores), has to be certainly emphasized.

Finally, the similarity in the experimentally deduced mechanism of inhibitory action between CPT and the two artesunate derivatives could be explained by the ability of the latter to recreate the key interactions, with amino acids Arg364 and Asp533, enabling CPT to inhibit the enzyme-DNA complex [13]. The computational study opens future prospects for the synthesis of novel inhibitors of human Topoisomerase I. In particular, in silico molecular docking and dynamics simulations showed the ability of these two new inhibitors to gain access to the enzyme-DNA interface and to position themselves in such a way that the bioactive part of the molecule occupies the interaction site of the known CPT inhibitor.

## 4. Materials and Methods

### 4.1. Data Preparation

The X-ray crystal structure of human DNA Topoisomerase I in complex with the poison camptothecin and covalent complex with 22 base pair DNA duplex (PDB ID: 1T8I) was used as the receptor for docking analysis [25]. Crystal ligands presented in the active site pocket of 1T8I were removed using PyMOL v. 2.5.0 (http://www.pymol.org, accessed on 10 October 2021). The structure of compounds **6** and **7** provided in Simplified molecular input line entry system (Smiles) format was converted in 3D coordinates using Open Babel software v. 2.3.2 [30]. The figures presented here are prepared using PyMol software (http://pymol.sourceforge.net, accessed on 10 October 2021).

### 4.2. In Silico Molecular Docking

In silico molecular docking is a computational method used to predict the formation of a molecular complex. The docking protocol was validated by redocking the crystal pose of CPT into the receptor. The same docking protocol was used to dock the two compounds. Both the compounds and the receptor were prepared for docking analysis using AutoDockTools v. 1.5.6 [27] and saved in pdbqt file format. The receptor was prepared by adding polar hydrogens and Kollman charges as partial charges. Gasteiger charges model was used to add partial charges to the two compounds. AutoDock Vina software [29] was used to perform docking analysis, obtaining 20 poses per compound. The docked poses were chosen based on the binding affinity and conformation. A grid box of 20 × 20 × 20 centered on the CPT was used as the search space.

### 4.3. Classical Molecular Dynamics Simulation

Classical molecular dynamics simulations were performed using Gromacs 2021.2 (https://gromacs.org/2021.2/.html, accessed on 15 November 2021) [31] with the *AMBER99* force field [32]. The topologies of the two compounds were generated using Acpype software [33] with AMBER atom types. The 1T8I-compound complexes obtained by the docking analysis were used as input for the molecular dynamics simulation. Both simulation complexes were centered in a box solvated with the TIP3 water model [34]. The solvated system was made electronically neutral by adding 18 sodium ions. Energy minimization of the complexes was performed via the steepest descent minimization algorithm with a maximum number of minimization steps of 50,000 and stopped when the maximum force was less than 1000.0 kJ/mol/nm. Potential energy was monitored before moving to the equilibration steps. After the energy minimization step an ensemble equilibration of number of particles, volume and temperature (NVT) was performed using V-rescale algorithm, for the duration of 1 ns, to maintain constant temperature at 300 K. Subsequently an ensemble equilibration of number of particles, volume and temperature (NPT) was performed using Berendsen barostat, for the duration of 2 ns, to bring the pressure to a constant value of 1 bar. Long-range electrostatics were calculated with the Particle Mesh Ewald method. As a result, the complexes were prepared for unrestrained 1 us production simulations with a time step of 2 fs and with coordinates saved every 10 ps.

### 4.4. Analysis of Non-Covalent Interactions

All the downstream bioinformatics analyses of the molecular dynamic simulation (RMSD, RMSF and ligand-recetor molecular interaction calculations) were performed on the high-performance computing systems of Cineca and provided by ELIXIR-IT HPC@CINECA call [35]. The analysis of noncovalent interactions between the two compounds and 1T8I was performed using the protein-ligand interaction profiler software, known as PLIP, choosing among the advanced options to tread the 22 bp of DNA as part of the receptor. Such software made it possible to detect salt bridges, hydrogen bonds, bridges mediated by water molecules, hydrophobic interactions and π-stacking interactions. The algorithm behind PLIP uses four steps to detect noncovalent interactions between a receptor and small ligands. The preparation step sees the addition of hydrogen atoms to the input structure and the extraction of the ligands along with their binding site. PLIP makes use of a blacklist to exclude preparation artifacts, modified amino acid residues, ions and water molecules. The second step sees the functional characterization of interacting groups, through the detection of hydrophobic atoms and hydrogen bond acceptor/donor atoms. In addition, PLIP searches for aromatic rings and charge centers in the structure provided as input. As for protein molecules, positive charges are assigned by PLIP to the azides of the side chains of arginine, histidine and lysine, while negative charges are assigned to the carboxyl groups of aspartic and glutamic acid. In the third step of the algorithm, the putative interaction groups are matched through the application of geometric criteria, in terms of angle and bond distance. Finally, the last filtering step is used to eliminate redundant interactions or interaction overlaps.

## Figures and Tables

**Table 1:** Molecular docking results for: (i) each of the new synthetized artemisinin-based derivatives (compounds **c0**–**c7**), (ii) the camptothecin (CPT), (iii) other compounds (CPT-derived) considered hTop1p inhibitors. For all the listed compounds the molecular docking simulation was performed with the CPT-deprived 1T8I complex by running the most used software for moleular docking, Autodock Vina.

Compound Name	2D Structure	Smiles Stringes	Binding Affinity (kcal/mol)	Reference
c0	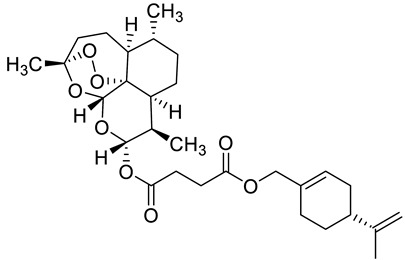	C[C@]1(OO2)O[C@](O[C@](OC(CCC(OCC3=CC[C@H](C(C)=C)CC3)=O)=O)([H])[C@@H]4C)([H])[C@]2([C@@]4([H])CC[C@H]5C)[C@@]5([H])CC1	−8.2	[12]
c1	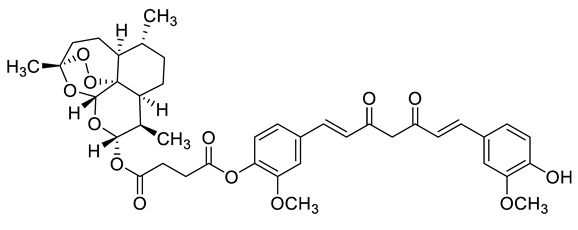	C[C@]1(OO2)O[C@](O[C@](OC(CCC(OC3=C(OC)C=C(/C=C/C(CC(/C=C/C4=CC(OC)=C(O)C=C4)=O)=O)C=C3)=O)=O)([H])[C@@H]5C)([H])[C@]2([C@@]5([H])CC[C@H]6C)[C@@]6([H])CC1	−7.7	[12]
c2	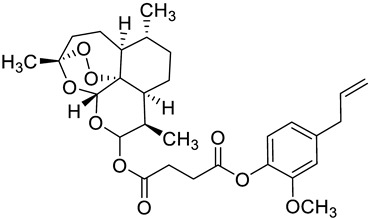	C[C@]1(OO2)O[C@](OC(OC(CCC(OC(C(OC)=C3)=CC=C3CC=C)=O)=O)[C@@H]4C)([H])[C@]2([C@@]4([H])CC[C@H]5C)[C@@]5([H])CC1	−7.7	[12]
c3	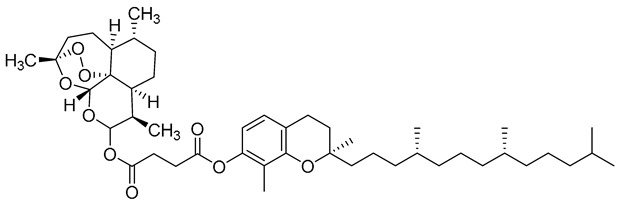	C[C@]1(OO2)O[C@](OC(OC(CCC(OC3=C©C4=C(CC[C@](CCC[C@H]©CCC[C@H]©CCCC©C)©O4)C=C3)=O)=O)[C@@H]5C)([H])[C@]2([C@@]5([H])CC[C@H]6C)[C@@]6([H])CC1	−7.0	[12]
c4	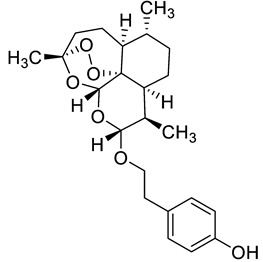	C[C@]1(OO2)O[C@](O[C@](OCCC3=CC=C(O)C=C3)([H])[C@@H]4C)([H])[C@]2([C@@]4([H])CC[C@H]5C)[C@@]5([H])CC1	−7.8	[12]
c5	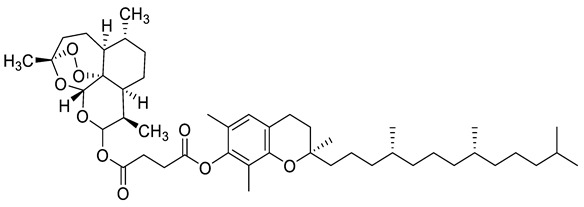	CC1=C(OC(CCC(OC([C@@H]2C)O[C@]3([H])O[C@](C)(OO4)CC[C@]5([H])[C@@]34[C@@]2([H])CC[C@H]5C)=O)=O)C(C)=CC6=C1O[C@@](CCC[C@H](C)CCC[C@H](C)CCCC(C)C)(C)CC6	−6.1	[12]
c6	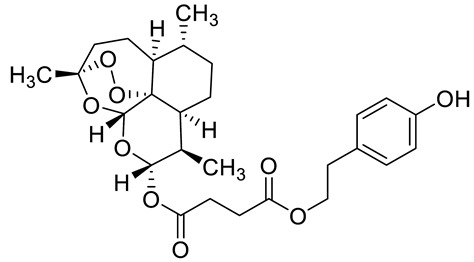	C[C@]1(OO2)O[C@](O[C@](OC(CCC(OCCC3=CC=C(O)C=C3)=O)=O)([H])[C@@H]4C)([H])[C@]2([C@@]4([H])CC[C@H]5C)[C@@]5([H])CC1	−9.1	[12]
c7	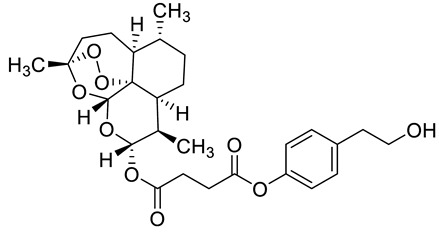	C[C@]1(OO2)O[C@](O[C@](OC(CCC(OC3=CC=C(CCO)C=C3)=O)=O)([H])[C@@H]4C)([H])[C@]2([C@@]4([H])CC[C@H]5C)[C@@]5([H])CC1	−8.7	[12]
CPT	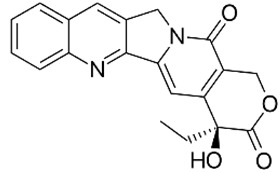	CCC1(C2=C(COC1=O)C(=O)N3CC4=CC5=CC=CC=C5N=C4C3=C2)O	−8.5	[7]
CPT21	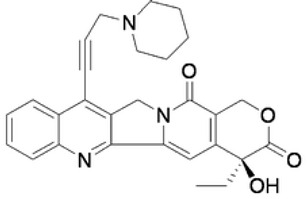	CC[C@@]1(C2=C(COC1=O)C(=O)N3CC4=C(C3=C2)N=C5C(=C4)C=CC=C5C#CCC6CCCNC6)O	−7.6	[22]
Topotecan	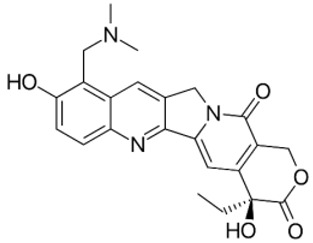	CC[C@@]1(C2=C(COC1=O)C(=O)N3CC4=CC5=C(C=CC(=C5CN(C)C)O)N=C4C3=C2)O	−8.7	[23]
SN-38	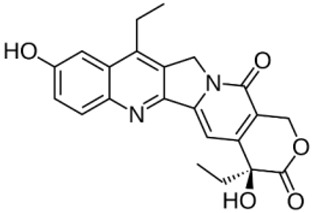	CCC1=C2CN3C(=CC4=C(C3=O)COC(=O)[C@@]4(CC)O)C2=NC5=C1C=C(C=C5)O	−8.1	[23]

**Table 2:** Ligand-receptor binding interactions per bond type over simulation time (1 µs, conformation sampling every 10 ps).

	Hydrogen Bonds	pi-Stacking Interactions	Hydrophobic Interactions	Water Bridges
Compound **c6**	100%	45.1%	98.4%	30.6%
Compound **c7**	99.8%	77.8%	99.5%	27.6%

**Table 3:** Percentage of the permanence of most relevant non-covalent interactions between the atoms of the compound **c6** and the amino acids or nucleotides of the receptor 1T8I.

	Glu356	Arg364	Lys425	Tyr426	DT9	DC111	DC112	DA113	DA114
O	-	-	10%	-	-	-	-	-	-
O1	-	-	-	98%	-	-	-	-	-
O2	-	-	-	-	-	-	-	93%	35%
O5	16%	-	-	-	-	-	10%	-	-
O6	-	73%	-	-	-	-	-	-	-
O8	-	-	-	-	-	14%	-	-	-
C13	-	-	-	-	56%	-	-	-	-

**Table 4:** Percentage of the permanence of most relevant non-covalent interactions between the atoms of the compound **c7** and the amino acids or nucleotides of the receptor 1T8I.

	Asn352	Arg364	Tyr426	Lys532	Asp533	DT9	DT10	DA113	DA114
O	-	-	18%	-	-	-	-	-	-
O1	15%	-	76%	-	-	-	-	-	-
O2	-	-	-	-	-	-	-	54%	51%
O3	-	-	-	-	-	-	-	45%	-
O6	-	11%	-	-	-	-	-	-	-
O8	-	22%	-	14%	19%	-	29%	-	-
C13	-	-	-	-	-	72%	-	-	-

## Data Availability

The data that supports the findings of this study are available within the article. In particular, for the results of molecular docking simulation, a project on figshare was created, and the corresponding data are available on the link: https://doi.org/10.6084/m9.figshare.21506178.v4 (accessed on 1 October 2022).
